# Differentiation of mucinous from non-mucinous pancreatic cyst fluid using dual-stained, 1 dimensional polyacrylamide gel electrophoresis

**DOI:** 10.1186/1559-0275-11-42

**Published:** 2014-12-01

**Authors:** John M Streitz, Michael T Madden, Wilmar Salo, Kirk P Bernadino, Joseph L Deutsch, John C Deutsch

**Affiliations:** Departments of Surgery, Essentia Health System/Duluth Clinic, University of Minnesota Medical School, Duluth, MN USA; Biomedical Sciences, Essentia Health System/Duluth Clinic, University of Minnesota Medical School, Duluth, MN USA; Statistics, University of Wisconsin, Madison, WI USA; Gastroenterology, Essentia Health System/Duluth Clinic, University of Minnesota Medical School, Duluth, MN USA

**Keywords:** Pancreatic cancer, Pancreatic cyst fluid, Biomarkers, Mucins, 1-D-PAGE

## Abstract

**Background:**

Pancreatic cysts are being increasingly identified in patients. Mucinous cysts have malignant potential whereas non-mucinous cysts do not. Distinguishing potentially malignant cysts from harmless ones by the characterization of cyst fluid contents remains a difficult problem. This study was undertaken to determine whether cyst fluid mucin glycoprotein analysis could differentiate mucinous from non-mucinous pancreatic cysts.

**Methods:**

Cyst fluid from 28 patients who underwent resection of a pancreatic cyst was used for the study. In each case the type of cyst was histologically identified. One dimensional SDS polyacrylamide gel electrophoresis (1D-SDS PAGE) was performed on cyst fluid samples. For the detection of the separated proteins, we employed a novel dual staining technique. The gel was first stained with periodic acid Schiff (PAS), a mucin histochemical stain followed by a secondary protein staining with Simply Blue Safestain (Invitrogen).

**Results:**

Visual scoring (based on the presence of mucins) gave a sensitivity of 95%, a specificity of 100%, a positive predictive value of 100%, and a negative predictive value of 88% for prediction of mucinous histology.

**Conclusions:**

One dimensional SDS polyacrylamide gel electrophoresis of pancreatic cyst fluid, followed by mucin (PAS) and protein (Simply Blue Safestain) staining, provides a means of concentrating and visualizing mucins, which allows the accurate differentiation of mucinous from non-mucinous histology in pancreatic cysts.

## Background

Pancreatic cysts are being increasingly identified by improved imaging methods, including endoscopic ultrasound (EUS), which allows cyst fluid sampling. Some mucinous cysts have malignant potential, whereas non-mucinous cysts do not. Surgical resection is often recommended for mucinous cystic lesions of the pancreas because of cancer risk [1, 2], but differentiating harmless cysts from potentially malignant ones remains difficult. Whether to resect or observe certain cysts is a common clinical problem owing to the inability to distinguish mucinous from non-mucinous cysts.

Pancreatic neoplasms with malignant potential such as mucinous cystadenoma, and intraductal papillary mucinous neoplasm (IPMN), can be distinguished to some extent, using cyst fluid analysis techniques, from cysts with no malignant potential, such as serous cystadenoma and pseudocyst. Cytology of cyst fluid showing dysplastic features is strongly predictive of mucinous neoplasia but is often falsely negative [3]. Clinical staining methods looking for mucin in the cyst fluid of pancreatic cysts have shown variable positivity but are considered unreliable [4, 5]. The cyst fluid carcinoembryeonic antigen (CEA) level has proven more reliable but is not a consistent positive predictor of mucinous histology by itself [6].

This research was undertaken to evaluate a pancreas cyst fluid characteristic that could reliably identify mucinous histology and therefore help to guide therapy. Mucinous cystic lesions of the pancreas are known to express mucins in their cyst fluid [7]. Clinical mucin staining often fails to identify mucins, either because of dilution, low level of expression, inhomogeneous distribution within the fluid sample or interference by other glycoproteins. Research has shown that mucins are uniformly found in the high molecular weight region (>250 K daltons) of a one dimensional, polyacrylamide gel [8, 9]. Using the technique of one dimensional, polyacrylamide gel electrophoresis (1D-SDS PAGE) we were able to separate mucins from smaller proteins within the fluid, concentrating mucins near the origin of the gel. The dual staining procedure effectively demonstrated the presence of mucins in the cyst fluid of mucinous cystic lesions.

## Results

Results of the stain scoring are shown in Table 1. None of the non-mucinous cysts showed staining for mucin, whereas all of the mucinous cystadenomas and 14 of the 15 IPMN specimens (93%) showed positive mucin staining. As a test for detecting mucinous neoplasms, these results yielded a sensitivity of 95%, a specificity of 100%, a positive predictive value of 100%, and a negative predictive value of 88%. Prediction intervals using a binomial model were calculated for the number of false negatives and false positives; under this model each count will be less than 19% of the sample size with a probability of 0.95.

**Table 1:** **Histologic diagnosis compared to stain scoring**

	Total	Stain scoring	
Histologic diagnosis	Number	Negative	Positive
*Non-mucinous cyst*			
Non-mucinous cyst	3	3	
Pseudocyst	4	4	
*Mucinous cyst*			
IPMN benign	6	1*	5
IPMN malignant	9		9
Mucinous cystadenoma	6		6

*False negative.

CEA Results (reported as ng/ml) are listed in Table 2. Using a CEA level of >200 ng/ml to indicate mucinous histology [10], CEA results had one false positive and two false negative results.

**Table 2:** **Carcinoembryonic Antigen (CEA) levels, compared to staining scoring**

		Mucin
Histologic diagnosis	CEA level (ng/ml)	Stain scoring
***Non-mucinous***		
P15 (cystic lymphangioma)	0	0 - Negative
Pan19 (serous microcystic adenona)	0	0 - Negative
P64 (lymphoepithelial cyst)	1576**	0 - Negative
Pan18 (pseudocyst)	0	0 - Negative
Pan21 (pseudocyst)	0	0 - Negative
Pan22 (pseudocyst)	0	0 - Negative
P39 (pseudocyst)	0	0 – Negative
***Mucinous***		
P10 (IPMN benign)	994	3 - Positive
P31 (IPMN benign)	106*	2 - Positive
P32 (IPMN benign)	878	1 - Positive
P43 (IPMN benign)	1011	0 -Negative
P46 (IPMN benign)	589	2 - Positive
P56 (IPMN benign)	598	1 - Positive
P4 (IPMN malignant)	555	2 - Positive
P7 (IPMN malignant)	438	1 - Positive
P41 (IPMN malignant)	988	2 - Positive
P55 (IPMN malignant)	589	2 - Positive
P57 (IPMN malignant)	1493	3 - Positive
P58 (IPMN malignant)	654	2 - Positive
P61 (IPMN malignant)	229	2 - Positive
P65 (IPMN malignant)	329	1 – Positive
P68 (IPMN malignant)	26*	1 – Positive
Pan16 (Mucinous cystadenoma)	1572	1 – Positive
P24 (Mucinous cystadenoma)	517	2 - Positive
P25 (Mucinous cystadenoma)	2253	3 - Positive
P40 (Mucinous cystadenoma)	1065	3 - Positive
P63 (Mucinous cystadenoma)	208	2 – Positive
P69 (Mucinous cystadenome)	5	2 – Positive

*False negative; **False positive.

Four samples did not reach the 5 ug level for protein loading. All of these were mucinous samples and had total protein content of 1.95 ug, 1.6 ug, 1.6 ug and 3.2 ug; they were all scored as positive for mucin, with respective protein scores of 1, 2, 2, and 2.

## Discussion

Diagnostic tests of pancreatic cyst fluid that can accurately predict cyst wall histology are needed in order to differentiate between harmless cystic lesions and premalignant, mucinous lesions that would require surgical intervention. Mucicarmine fluid staining for extracellular mucin is a weak predictor of mucinous histology, with 38% to 55% of mucinous cystic lesions testing positive, as well as 40% of pseudocysts [7, 11]. It has a reported positive predictive value of 61% in one study [6]. Cytology of cyst fluid is strongly predictive of mucinous histology when positive, but is an insensitive test. [6, 10, 12]. Cyst fluid CEA level has shown sensitivity when present in high amounts [10, 12], but its positive predictive value as an independent test in a large cooperative study was 79% [10] and has been reported to be as low as 51% by others [6]. Furthermore, investigators use different levels of CEA to predict mucinous histology in different reports, depending on whether or not the goal is sensitivity, specificity or accurate. Exhaustive investigation into the entire cyst fluid proteome using mass spectrometry has so far yielded no biomarkers any more useful than the CEA level [5, 13]. 1-D SDS PAGE of pancreatic cyst fluid, utilizing a single protein specific stain (SYPRO Ruby fluorescence), has been employed by others with no demonstrable diagnostic value [8]. Specific cyst fluid (non-mucinous) glycoproteins have shown some promise as biomarkers for mucinous histology, with independent sensitivity in one study as high as 78% [8].

The 1-D SDS PAGE/mucin staining method we have described, shows a high sensitivity, specificity, positive predictive value, and negative predictive value for detecting mucinous neoplasms. This is due to the separation of mucin glycoproteins from other proteins and their concentration on the gel.

Protein electrophoresis is commonly employed in clinical laboratories. This makes it possible that this method could be converted to a diagnostic test for clinical use.

The visual scoring of mucin content is a weakness in the analysis. The use of densitometry or quantitation of the mucin glycoprotein levels might allow a more accurate evaluation. Nonetheless, visual scoring methods, such as the one that we employed, are often used in clinical medicine. The Her2 Neu staining for breast cancer, which is visually scored 0 to 3+ based on an estimate of immunohistochemistry staining of cell membranes, is considered a reliable clinical tool. CEA levels continue to be the used as the primary clinical biomarker of mucinous cystic lesions. While CEA level was not part of our primary data analysis, the results are included as an interesting clinical correlation.

The expanding field of glycoproteomics makes use of mass spectrometry (MS) to analyze complex biologic fluids such as pancreatic cyst fluid. It requires the enrichment of the mucin glycoprotein content in the fluid to allow MS to identify peptides expressed in low concentrations. A number of methods have been used to achieve this enrichment, including lectin affinity chromatography, hydrophilic solid phase extraction, boric acid, and hydrazide chemistry [14, 15]. The 1-D SDS PAGE/ dual staining technique presented herein displays the ability to effectively enrich the mucin glycoprotein in the samples, which should aid in further proteomic analyses of these cysts.

Our ongoing research to identify specific mucins present in pancreatic cyst fluid, using MS, could enable more exact differentiation between the various mucinous and non-mucinous cyst types. Particular mucins identified within the cyst fluid may prove to be useful as biomarkers for mucinous histology.

## Conclusion

Pancreatic cyst fluid, which often shows a high degree of variation from one sample to the next, is difficult to analyze. The technique of one dimensional SDS polyacrylamide gel electrophoresis of the pancreatic cyst fluid, followed by dual staining for mucin (PAS) and protein (Simply Blue Safestain), provides a means of concentrating and visualizing the mucins. This allows the accurate differentiation of mucinous from non-mucinous histology in pancreatic cysts.

## Methods

Samples: Between November 2005 and January 2011, twenty-eight fluid samples were obtained from patients with surgically excised cystic pancreatic lesions, under an IRB approved protocol for the purpose of this research. Each lesion had an unequivocal histological diagnosis on postoperative pathologic examination, as listed in Table 3. All IPMN samples categorized as benign displayed low-grade dysplasia by definition. Those cases of IPMN displaying high-grade dysplasia were categorized as malignant. Fluid samples were obtained by a single investigator (JMS) in the course of operative resection, either from the in situ specimen or immediately upon removal of the cyst from the patient. The fluid samples were frozen immediately within dry ice in plastic containers and then transferred directly to a minus 70 degree freezer at the conclusion of the operation. Pseudocysts were treated with enteric drainage, but a portion of the pseudocyst cyst wall was sent for histology, and all of these cysts resolved following treatment.

**Table 3:** **Postoperative histologic diagnosis**

Histologic diagnosis	Number	Percent
*Non-mucinous cyst*	*7*	*25%*
Serous microcystic adenoma	1	3.6%
Cystic lymphangioma	1	3.6%
Lymphoepithelial cyst	1	3.6%
Pseudocyst	4	14.3%
*Mucinous cyst*	*21*	*75%*
Mucinous cystadenoma	6	21.4%
IPMN benign	6	21.4%
IPMN malignant	9	34.2%

### Sample preparation

Each sample was thawed and vortexed to ensure thorough mixing. An aliquot was removed and the sample refrozen. Protein content of the fluid was determined using the Pierce BCA protein assay kit (Thermo Scientific, Rockford IL), and 5 ug of protein used, as this is a commonly employed amount to allow clear separation of protein bands during electrophoresis. In samples where the protein concentration was too low to reach the 5 ug level (N = 4 of 28), the maximum volume for gel loading (6.5 ul) was used.

### Electrophoresis

Cyst fluid samples were run on a 4-12% NuPage Novex Bis-tris minigels (Invitrogen, Carlsbad CA) with MES (2-morpholinoethanesulfonic acid) running buffer containing 50 mM MES, 50 mM Tris Base, 0.1% SDS, 1 mM EDTA at pH 7.3.

Samples were run under reducing conditions, prepared by combining the sample with 2.5 ul NuPage sample buffer (4×), 1ul NuPage reducing agent (10×), and then brought to a final volume of 10 ul. Samples were heated to 70 degrees C for 10 minutes (as per manufactures protocol), vortexed, spun down and loaded on the gel. The gel was then run at 200 volts constant for 35 minutes.

### Staining

**Figure 1:**
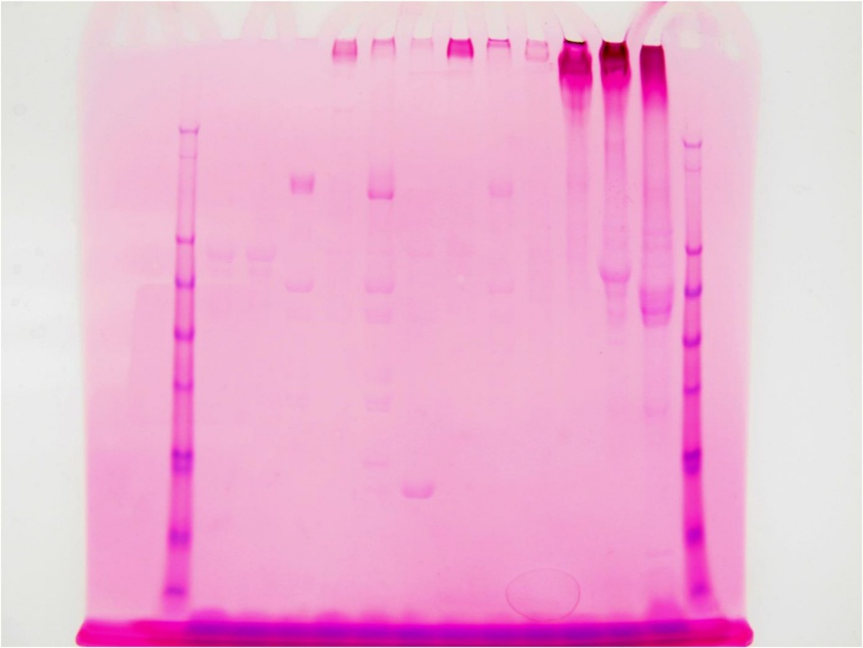
**1-D SDS PAGE; primary PAS glycoprotein staining of representative pancreatic cyst fluid samples.** (Direction of travel on the gel is downward).

Primary staining was done with periodic acid-Schiff (PAS). Following electrophoresis the gel was immersed in 12.5% trichloracetic acid for 20 minutes, then 1% periodic acid for 25 minutes in the dark. It was then immersed in Schiff’s fuchsin-sulfite reagent (Sigma-Aldrich, St. Louis MO) for 15 minutes in the dark, washed in 0.5% metabisulfate twice and then washed in water until the gel destained. The gel was then photographed. As seen in Figure 1, results showed red staining of the glycoproteins.

**Figure 2:**
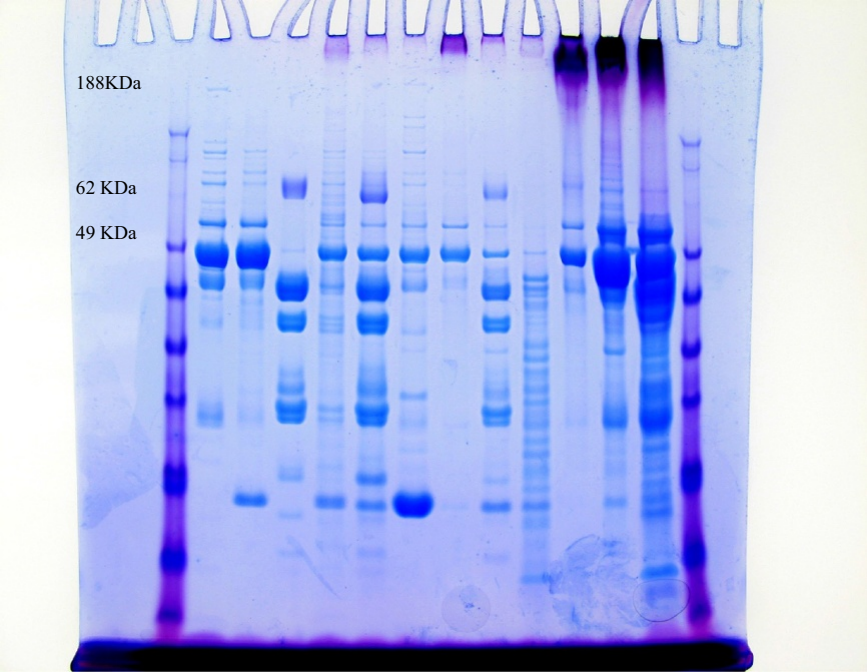
**1-D SDS PAGE; dual stained pancreatic cyst fluid samples (PAS and SimplyBlue).**

**Figure 3:**
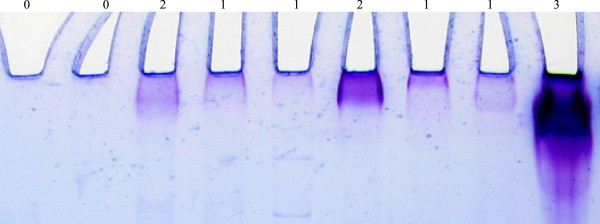
**Close up of representative lane origins and the purple, dual stained bands used for scoring.** Visual staining scores are above each specimen.

The secondary stain utilized the SimplyBlue SafeStain (Invitrogen, Carlsbad CA). Following PAS staining the gel was placed in 100 ml of water and microwaved to almost boiling; the water was then discarded, with this process repeated twice. The gel was then covered with SimplyBlue SafeStain and microwaved about 1.5 minutes, washed in 100 ml water and then 20 ml of 20% NaCl was added for 5 minutes. The gel was then photographed. The results showed blue staining of all proteins and purple (red primary and blue secondary) staining of glycoproteins (Figures 2 and 3).

### Glycoprotein stain scoring

The dual stained gels were visually assessed by an evaluator blinded to the cyst fluid histology. The degree of mucin staining was accessed immediately downstream of the well. Staining at the 0 level was designated negative for mucin. Any detectable mucin staining was designated as positive and given a +1, +2 or +3 valuation (Figures 2 and 3).

### Carcinoembryonic Antigen (CEA)

The carcinoembryonic antigen (CEA) in each fluid sample was determined using an enzyme-linked immunosorbent assay (ELISA) kit for CEA (ALPCO, Salem NH) as per manufacturer’s protocol.

### Statistical analysis

Binomial distribution prediction intervals were calculated for the false positive and false negative rates using the technique of Krishnamoorthy [16].
